# Association of smoking and osteoarthritis in US (NHANES 1999–2018)

**DOI:** 10.1038/s41598-023-30644-6

**Published:** 2023-03-08

**Authors:** Senbo Zhu, Lichen Ji, Zeju He, Wei Zhang, Yu Tong, Junchao Luo, Zheping Hong, Yin Zhang, Dongsheng Yu, Qiong Zhang, Qing Bi

**Affiliations:** 1 Center for Rehabilitation Medicine, Department of Orthopedics, Zhejiang Provincial People’s Hospital (Affiliated People’s Hospital, Hangzhou Medical College), Hangzhou, 310014 Zhejiang China; 2 Department of Orthopedics, The Second Affiliated Hospital and Yuying Children’s Hospital of Wenzhou Medical University, Wenzhou, 325027 Zhejiang China; 3 Center for Operating Room, Department of Nursing, Zhejiang Provincial People’s Hospital (Affiliated People’s Hospital, Hangzhou Medical College), Hangzhou, 310014 Zhejiang China

**Keywords:** Diseases, Health care

## Abstract

Little is currently known about the effect of smoking on osteoarthritis (OA). This study aimed to investigate the relationship between smoking and OA in the United States (US) general population. Cross-sectional study. Level of evidence, 3. 40,201 eligible participants from the National Health and Nutrition Examination Survey 1999–2018 were included and divided into OA and non-arthritis groups. Participants demographics and characteristics were compared between the two groups. Then the participants were divided into non-smokers, former smokers, and current smokers based on their smoking status, also demographics and characteristics among the three groups were compared. Multivariable logistic regression was used to determine the relationship between smoking and OA. The current and former smoking rate in the OA group (53.0%) was significantly higher than that in the non-arthritis group (42.5%; *p* < 0.001). Multivariable regression analysis including body mass index (BMI), age, sex, race, education level, hypertension, diabetes, asthma and cardiovascular disease showed that smoking was an association for OA. This large national study highlights a positive association between smoking and OA prevalence in the general US population. It is necessary to further study the relationship between smoking and OA in order to determine the specific mechanism of smoking on OA.

## Introduction

Osteoarthritis is a common disease in the elderly, characterized by progressive degeneration of articular cartilage, subchondral bone changes, and osteophyte formation^1^. OA is estimated to affect more than 240 million people worldwide, with more than 32 million estimated in the United States, resulting in a significant public health burden^2^. Based on data from the National Health Interview Survey, recently in the United States, there are approximately 14 million people who have symptomatic knee osteoarthritis of which approximately 3 million ethnic/racial minorities^3^. Thus, identifying potentially modifiable protective or risk factors may lead to the development of available strategies to delay the progression of OA and reduce the public health burden.

It is well-established that smoking is linked to chronic diseases such as diabetes, cardiovascular disease, and cancer. It is also a recognized risk factor for many chronic musculoskeletal diseases, including rheumatoid arthritis, degenerative disc disease, and low back pain. However, smoking is reportedly negatively associated with ulcerative colitis and Parkinson's disease. The relationship between smoking and OA has not been established^4^. Many clinical studies in different regions and populations have shown that smoking is negatively associated with the incidence of OA^1,5–7^. There is some evidence suggesting that the BMI of non-smokers is usually higher than that of smokers, leading to a high OA prevalence^8,9^. However, some scholars have raised doubts on the protective effect of smoking on OA observed in some epidemiological studies due to selection bias, especially hospital selection bias^10^. In a cross-sectional research of nationally representative data from South Korea, no association was found between direct smoking and previous smoking with the prevalence of OA^11^. A cross-sectional study from Denmark also found no association between current smoking and knee OA prevalence^12^. Given that the relationship between smoking and OA remains unclear, the results vary widely among different populations and regions. There are still contradictions and inconsistencies in the relationship between smoking and OA risk. In addition, few studies have examined in detail the relationship between current and former smokers and OA.

Therefore, this study aimed to explore the relationship between smoking and previous smoking on OA among US adults using national cross-sectional data. It also offered a valuable opportunity for assessing the potential of pooling OA preventive health information.

## Methods

### Study population data

**Figure 1:**
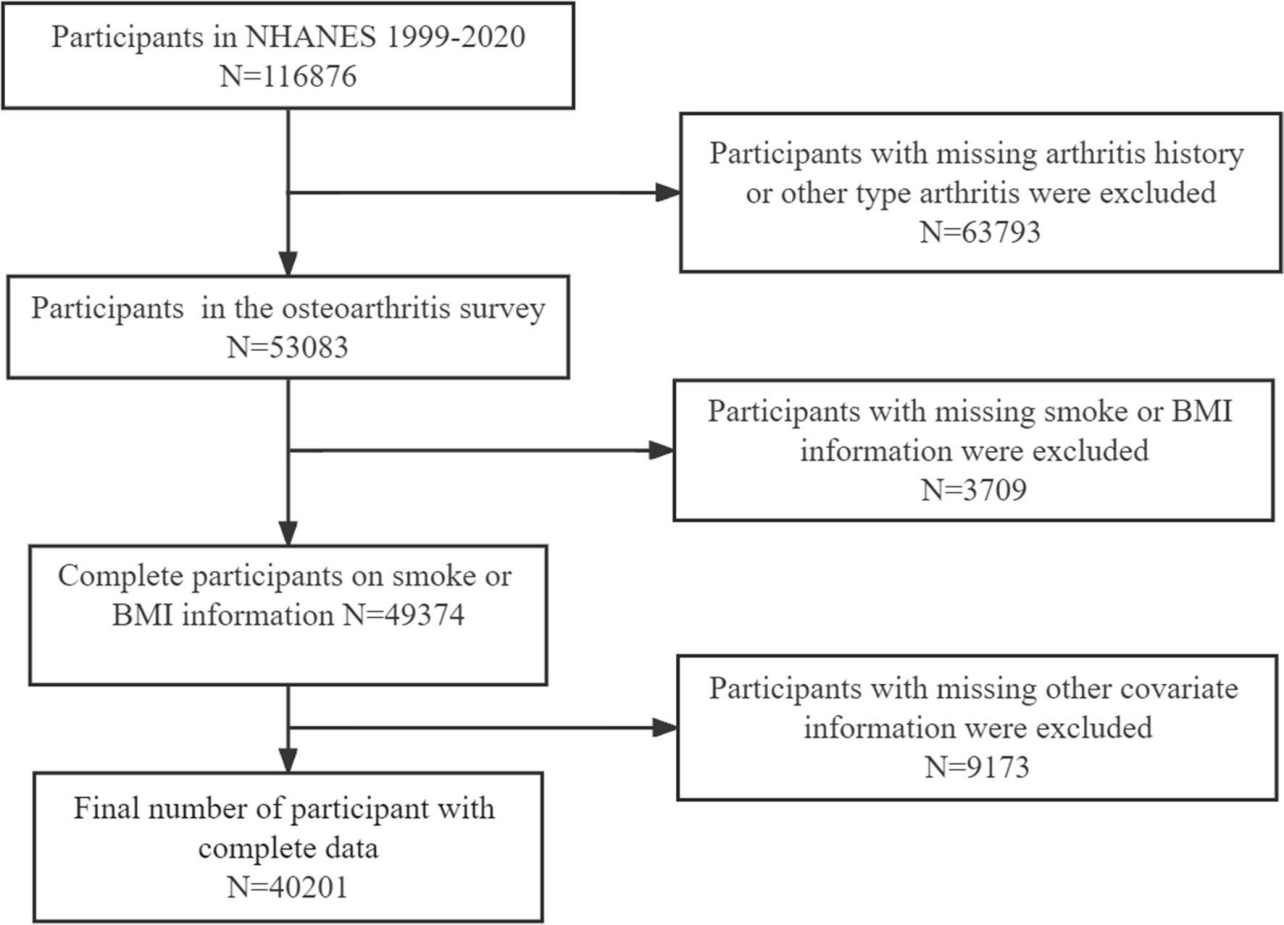
Flowchart of study participants.

Data from ten discrete 2-year cycles (1999–2000 to 2017–2018) of the continuous National Health and Nutrition Examination Survey were used to examine the association of OA with smoking and BMI in US adults. NHANES (http://www.cdc.gov/nchs/nhanes.htm) is a national cross-sectional health survey in the US that collects laboratory, imaging, and radiological data in addition to health interviews and examination data^13^. Importantly, the Center for Disease Control (CDC) and Prevention uses a complex multi-stage probabilistic sampling design to examine a nationally representative sample across the country every 2 years. The NHANES study protocol has been approved by the Ethics Review Board of the National Center for Health Statistics (NCHS) Research, and all adult participants provided written informed consent. All studies were carried out in accordance with the Declaration of Helsinki. Details on Institutional Review Boards of the CDC and NCHS are available at (http://www.cdc.gov/nchs/nhanes/irba98.htm). Given the thoroughness of its methodology, NHANES data have been widely used to assess risk factors and prevalence of many diseases^14^. In this study, 116,876 participants older than or equal to 20 were selected from 1999 to 2018 NHANES (As shown in Fig. 1). Participants without OA data and with other types of arthritis were excluded. Participants with missing smoking, BMI, and other covariate data were also excluded.

### OA criteria

The NHANES Medical Conditions Questionnaire is usually based on the "Medical condition" questionnaire of the American National Health Interview survey, and the OA status is investigated and collected. Participants are asked, "Has a doctor or other health professional ever told (you/s/he) that (you/s/he) . . .had arthritis?" If the answer to that question is "yes" the next question is "What type of arthritis was it?". Based on the different responses to these two questions, participants were divided into an OA group (solely had OA), other arthritis participants (which were excluded from further analysis) and no-arthritis group. Self-reported doctor-diagnosed OA is the most predominantly used case definition in epidemiological studies^14^. The agreement between self-reported OA and clinically confirmed OA was 81% in a previous study^15^.

### Assessment of smoking volume, and other covariates

Variables associated with clinical characteristics were collected through physical examinations and self-reported questionnaires. Participants were divided into three categories based on their smoking status: never smokers (smoked less than 100 cigarettes in life), past smokers (smoked more than 100 cigarettes in life and smoke not at all now), and current smokers (smoked moth than 100 cigarettes in life and smoke some days or every day). The BMI (kg/m^2^) was recorded for all examinees by a trained examiner in the mobile examination center. According to the National Institutes of Health (NIH) guidelines^16^, BMI was divided into four grades: underweight (< 20.0 kg/m^2^), normal weight (20.0–24.9 kg/m^2^), overweight (25.0–29.9 kg/m^2^), and obese (≥ 30.0 kg/m^2^). Cardiovascular disease (CVD) included coronary heart disease, congestive heart failure, heart attack, stroke, and angina. Other covariates included discrete variables (sex, race/ethnicity, education, hypertension, diabetes mellitus (DM), asthma and marital status) and continuous variables (age, poverty status (family income to poverty ratio, PIR).

### Statistical analyses

Data were processed by SPSS version 22.0 and R version 4.1.3. The Chi-square test was used to compare the constituent ratios of each group. Continuous variables were compared using Student's t-test or one-way analysis of variance (ANOVA), followed by multiple post-hoc comparisons using least significant difference (LSD), selected based on Levene statistics in the homogeneity of variance test. Variables with a *p*-value < 0.25 during univariate analysis were included in multivariable logistic regression analysis^17^. After adjusting for all covariables, logistic regression was performed to assess the association between smoking and OA. A *p*-value < 0.05 (two-sided) was statistically significant.

## Results

### Characteristics of participants

Participants younger than 20 years of age were excluded based on the epidemiological characteristics of OA. The prevalence of OA was 12.49% (n = 5022/40,201). The demographics and characteristics of participants in this study are shown in (Table 1).

**Table 1:** Demographics and characteristics of study participants from NHANES 1999–2018.

Characteristic	Osteoarthritis N = 5022 (12.49%)	Non-arthritis N = 35,179 (87.51%)	*p* value
Smoke, N (%)			< 0.001^a^
Never	2361 (47.0 1)	20223 (57.49)	
Ever	1847 (36.78)	7572 (21.52)	
Now	814 (16.21)	7384 (20.99)	
Age, year, N (%)			< 0.001^a^
20–29	62 (1.23)	7503 (21.33)	
30–39	210 (4.18)	7318 (20.80)	
40–49	469 (9.34)	6915 (19.66)	
50–59	901 (17.94)	5142 (14.62)	
60–69	1430 (28.47)	4431 (12.60)	
70–79	1146 (22.82)	2431 (6.91)	
≥ 80	804 (16.01)	1439 (4.09)	
Mean ± SD	63.92 ± 13.31	45.45 ± 16.97	< 0.001^b^
Gender, N (%)			< 0.001^a^
Male	1821 (36.26)	18,481 (52.53)	
Female	3201 (63.74)	16,698 (47.47)	
BMI, kg/m^2^, N (%)			< 0.001^a^
< 20 (underweight)	118 (2.35)	1761 (5.0)	
20–24.9 (normal weight)	915 (18.22)	9494 (26.99)	
25.0–29.9 (overweight)	1636 (32.58)	12,023 (34.18)	
> 30.0 (obese)	2353 (46.85)	11,901 (33.83)	
Mean ± SD	30.86 ± 7.64	28.51 ± 6.57	< 0.001^b^
Race, N (%)			< 0.001^a^
Mexican American	407 (8.10)	6318 (17.96)	
Non-hispanic black	744 (14.81)	7272 (20.67)	
Non-hispanic white	3227 (64.26)	14,476 (41.15)	
Other hispanic	285 (5.68)	3027 (8.60)	
Other race including multiracial	359 (7.15)	4086 (11.61)	
Education, N (%)			< 0.001^a^
Under high school	1002 (19.95)	8208 (23.33)	
High school or equivalent	1169 (23.28)	7964 (22.64)	
College graduate or above	2851 (56.77)	19,007 (54.03)	
PIR, N (%)			< 0.001^a^
< 1	747 (14.87)	6903 (19.62)	
1–3.5	2527 (50.32)	16,787 (47.72)	
≥ 3.5	1748 (34.81)	11,489 (32.66)	
Mean ± SD	2.74 ± 1.60	2.60 ± 1.63	< 0.001^b^
Marital, N (%)			< 0.001^a^
Married/Living with partner	2949 (58.72)	21,636 (61.50)	
Never married	340 (6.77)	7295 (20.74)	
Separated/Divorced/Widowed	1733 (34.51)	6248 (17.76)	
Hypertension, N (%)			< 0.001^a^
Yes	3025 (60.23)	9586 (27.25)	
No	1997 (39.77)	25,593 (72.75)	
Diabetes, N (%)			< 0.001^a^
Yes	1086 (21.62)	3467 (9.86)	
No	3936 (78.38)	31,712 (90.14)	
CVD, N (%)			< 0.001^a^
Yes	1140 (22.70)	2447 (6.96)	
No	3882 (77.30)	32,732 (93.04)	
Asthma, N (%)			< 0.001^a^
Yes	952 (18.96)	4210 (11.97)	
No	4070 (81.04)	30,969 (88.03)	

^a^means Chi-square test, ^b^means Student's t-test.

We found that OA was more common in older women (age ≥ 50), the average age of the OA group was significantly higher than the non-arthritis group (63.92 ± 13.31 vs. 45.45 ± 16.97, *p* < 0.001), and the proportion of females with OA was higher than males (63.74% vs. 36.26%). The proportion of obese patients in the OA group was significantly higher than in the non-arthritis group (46.85% vs. 33.83%), and the proportion of non-Hispanic White also increased significantly (64.26% vs. 41.15%). At the same time, OA was associated with a higher level of education and annual household income. Besides diabetes, the prevalence of other comorbidities in the OA group was significantly higher than in the non-arthritis group (*p* < 0.001). Most importantly, the proportions of non-smokers and smokers in the OA group were lower than in the non-arthritis group, but the proportion of former smokers was significantly higher in the OA group (36.78% vs. 21.52%, *p* < 0.05).

### Characteristics of participants by smoking status

The 40,201 participants were divided into never-smokers (n = 22,584), former smokers (n = 9419) and current smokers (n = 8198). The characteristics of 40,201 participants are shown in (Table 2).based on their smoking status. The average age of former smokers was the highest, and the average age of current smokers was the youngest (56.08 ± 17.12 vs. 42.96 ± 15.07 vs. 46.03 ± 17.59, *p* < 0.001). Never-smokers were predominantly women (57.59%). Compared with never-smokers, former smokers had a higher BMI, and current smokers had a lower BMI (28.89 ± 6.87 vs. 29.38 ± 6.43 vs. 27.90 ± 6.71, *p* < 0.001). This finding suggests that smoking may lead to a decrease in BMI, and quitting smoking increases BMI. Current smokers were associated with significantly lower levels of education, family income, and single status (including separated, divorced, widowed, and not being married, *p* < 0.001). In addition to asthma (smoking is considered an important risk factor) and CVD, other underlying diseases: hypertension, diabetes, smokers had significantly lower rates than non-smokers and quit smoking people (*p* < 0.001). Quit smoking people also had a higher prevalence of comorbidities than current smokers, which may be attributed to the fact that past smokers quit smoking after being diagnosed with comorbidities. The incidence of OA in the smoking group was 2.089 times higher than in the non-smoking group.

**Table 2:** Characteristics of participants based on smoking status.

Characteristic	Smoking status			*p* value
	Never	Ever	Now	
N	22,584	9419	8198	
Age, years, N (%)				< 0.001^a^
20–29	4887 (21.64)	790 (8.39)	1888 (23.03)	
30–39	4496 (19.91)	1139 (12.09)	1893 (23.09)	
40–49	4260 (18.86)	1413 (15.00)	1711 (20.87)	
50–59	3187 (14.11)	1564 (16.60)	1292 (15.76)	
60–69	2850 (12.62)	2030 (21.55)	981 (11.97)	
70–79	1709 (7.57)	1516 (16.10)	352 (4.29)	
≥ 80	1195 (5.29)	967 (10.27)	81 (0.99)	
Mean ± SD	46.03 ± 17.59 c*** d***	56.08 ± 17.12 e***	42.96 ± 15.07	< 0.001^b^
Sex, N (%)				< 0.001^a^
Male	9578 (42.41)	5841 (62.01)	4884 (59.58)	
Female	13,006 (57.59)	3578 (37.99)	3314 (40.42)	
BMI, kg/m^2^, N (%)				< 0.001^a^
< 20 (underweight)	1010 (4.47)	249 (2.64)	620 (7.56)	
20–24.9 (normal weight)	5941 (26.31)	2023 (21.48)	2445 (29.82)	
25.0–29.9 (overweight)	7506 (33.24)	3548 (37.67)	2605 (31.78)	
> 30.0 (obese)	8127 (35.99)	3599 (38.21)	2528 (30.84)	
Mean ± SD	28.89 ± 6.87 c*** d***	29.38 ± 6.43 e***	27.90 ± 6.71	< 0.001^b^
Race, N (%)				< 0.001^a^
Mexican american	4095 (18.13)	1510 (16.03)	1120 (13.66)	
Non-hispanic black	4747 (21.02)	1318 (13.99)	1951 (23.80)	
Non-hispanic white	8624 (38.19)	5140 (54.57)	3939 (48.05)	
Other hispanic	2073 (9.18)	701 (7.44)	538 (6.56)	
Other race including multiracial	3045 (13.48)	750 (7.96)	650 (7.93)	
Education, N (%)				< 0.001^a^
Under high school	4514 (19.99)	2217 (23.54)	2479 (30.24)	
High school or equivalent	4549 (20.14)	2141 (22.73)	2443 (29.80)	
College graduate or above	13,521 (59.87)	5061 (53.73)	3276 (39.96)	
PIR, N (%)				< 0.001^a^
< 1	3867 (17.12)	1347 (14.30)	2436 (29.71)	
1–3.5	10,506 (46.52)	4654 (49.41)	4154 (50.67)	
≥ 3.5	8211 (36.36)	3418 (36.29)	1608 (19.61)	
Mean ± SD	2.75 ± 1.65 c* d***	2.79 ± 1.60 e***	2.05 ± 1.63	< 0.001^b^
Marital, N (%)				< 0.001^a^
Married/Living with partner	14,012 (62.04)	6295 (66.83)	4278 (52.18)	
Separated/Divorced/Widowed	3892 (17.23)	2201 (23.37)	1888 (23.03)	
Never married	4680 (20.72)	923 (9.80)	2032 (24.79)	
Hypertension, N (%)				< 0.001^a^
Yes	6445 (28.54)	3954 (41.98)	2212 (26.98)	
No	16,139 (71.46)	5465 (58.02)	5986 (73.02)	
Diabetes, N (%)				< 0.001^a^
Yes	2327 (10.30)	1557 (16.53)	669 (8.16)	
No	20,257 (89.70)	7862 (83.47)	7529 (91.84)	
CVD, N (%)				< 0.001^a^
Yes	1445 (6.40)	1470 (15.61)	672 (8.20)	
No	21,139 (93.60)	7949 (84.39)	7526 (91.80)	
Asthma, N (%)				< 0.001^a^
Yes	2729 (12.08)	1244 (13.21)	1189 (14.50)	
No	19,855 (87.92)	8175 (86.79)	7009 (85.50)	

^a^means Chi-square test, ^b^means one-way ANOVA test, ^c,d,e^post-hoc comparisons using LSD between Never and Ever, Never and Now, and Ever and Now, respectively. *p < 0.05, **p < 0.01 ***p < 0.001.

### Association between OA and smoking

**Figure 2:**
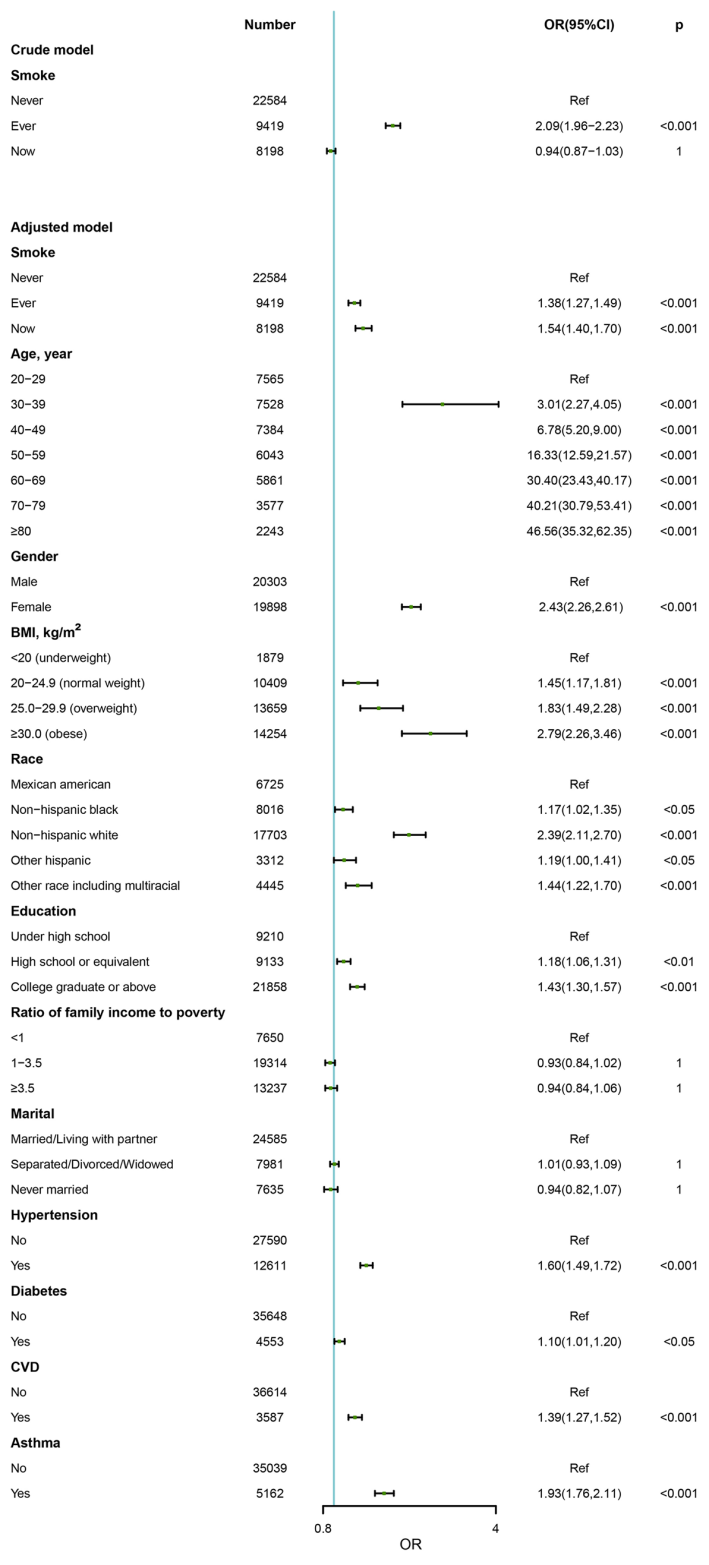
Logistic regression analysis of smoke for OA in participants ≥ 20 years old in NHANES (1999–2018).

Figure 2 shows the crude and adjusted odds ratios for the association between OA and smoking. In the crude model, the smoking status was statistically significantly associated with OA as a categorical variable. The incidence of OA in the former smoker group was 2.09 times higher than in the non-smoker group (OR = 2.09, 95%CI 1.96–2.23, *p* < 0.001). However, there was no significant difference between the smoker and non-smoker groups (OR = 0.94, 95%CI 0.87–1.03, *p* = 1). After adjusting for age, gender, BMI, race, education, PIR, marital state, hypertension, diabetes, CVD, asthma, both current smoker and former smoker groups were associated with increased risk of OA compared to the non-smoker group (OR = 1.54, 95%CI 1.40–1.70, *p* < 0.001 and OR = 1.38, 95%CI 1.27–1.49, *p* < 0.001, respectively).

## Discussion

Herein, we conducted a comprehensive study of OA using a large multi-year sample from NHANES 1999–2018. Indeed, previous knee OA studies on the NHANES database have been published in the literature, but one study was too old (being published in the 1970s), with a small number of participants and only cross-sectional associations between knee OA and various putative risk factors were explored^18^, and some studies did not focus on the relationship between smoking and OA^19–21^. The strength of this research is the numerous OA patients identified from a US population-based cross-sectional cohort. Another advantage is that, since they are acquired in the community, a lack of selection bias in exposure is assumed. Accordingly, we sought to explore the association between smoking and quitting smoking and OA. In the crude model, quitting smoking has a positive correlation with OA, while smoking has no correlation with OA. However, in our multivariable analysis model, which included variables measured in NHANES such as age, sex, BMI, and underlying disease to minimize the possibility of spurious association, we substantiated that current and past smokers had a higher risk of OA. The increased risk of OA attributed to smoking was not decreased significantly after quitting smoking (OR = 1.54, 95%CI 1.40–1.70, *p* < 0.001 and OR = 1.38, 95%CI 1.27–1.49, *p* < 0.001, respectively), continuing to smoke was associated with a greater risk of OA than in former smokers. Our study points out that high BMI and advanced age are significant risk factors for OA, and that women are at higher risk than men, which is consistent with previous reports^22,23^. In addition, our study shows that education level and underlying diseases such as hypertension and diabetes are also related to OA.

Large cohort studies based on the general population of the United Kingdom have shown that a weight gain of 4–5 kg after 12 months of smoking cessation may lead to increased knee degenerative disease^8^. Consistently, cohort studies in the Korean population have shown that a decrease in BMI caused by smoking may account for the protective effect of smoking against OA^1^. In accordance with previous studies, in this study, we found that an increase in BMI in former smokers and smoking led to a decrease in BMI. Among smokers, the proportion of overweight and obese people is 63.6%, which is the lowest among the three groups; among ex-smokers, the proportion of overweight and obese people is 75.9%, which is the highest among the three groups; among non-smokers, the proportion of overweight and obese people is 69.2%, the difference was statistically significant. However, after adjusting for BMI, a confounding factor, we still found a positive association between smoking and OA.

Smoking has been documented to exert heterogeneous effects on OA in different countries and regions. For example, smoking was found to be a protective factor against joint replacement with severe knee OA in a Singaporean Chinese cohort^5^. A cohort study on a Rotterdam population further showed that smoking was a risk factor for OA^24^. The reliability of many studies that concluded that smoking is a protective factor for OA was questioned due to study design bias. Indeed, hospital-based studies represent a significant source of bias. Smoking is more likely to cause health problems, which led to more non-osteoarthritis patients who smoke being included in the study^10^. It is widely established that the U.S. population is dominated by non-Hispanic whites. During multivariable regression analysis, the risk of OA in non-Hispanic whites was 2.39 times higher than in Mexican Americans. We suspect that the effects of smoking on different ethnic groups may lead to regional variations in studies. A multicenter trial in 2022 found an increased risk of gastrointestinal metaplasia (GIM) among Hispanics born outside the United States, but not among Hispanics born in the United States^25^. This finding suggests that members of the same ethnic group, born and living in different areas, may have different susceptibility to the same risk factors. Accordingly, the effect of smoking on OA varies across countries and regions.

At present, the exact mechanism between smoking and OA is poorly understood. Interestingly, studies have shown that nicotine alters 19 proteins, including several proteases and cytokines, in models of joint inflammation, including increased nicotine secretion of matrix metalloproteinase 1 and increased secretion of two proposed markers of OA, chitinase 3-like protein 1 fibronectin. Indeed, it is essential to increase awareness of the risk of OA, prevent its occurrence and improve the quality of life of this patient population. Current evidence suggests that age is the most significant risk factor for OA after age 30. In addition, the risk of OA was also significantly correlated with BMI, gender and race. Our findings suggest that people should reduce their smoking to reduce their risk of OA. In addition smoking causes a variety of harmful effects of carcinogens and chemicals.

The strengths of this study included its large-scale nature, the use of national cohort data, and the absence of selection bias. However, although NHANES is a representative sample of the general population in the United States, this is a cross-sectional study, which requires further validation of the effect of smoking on OA in prospective studies. In addition, a questionnaire was used to determine whether the participants were OA patients. Although the response rate was 81%, the gold standard for determining OA was imaging, and no physical examination was performed to determine whether participants had OA. And the absence of other covariates, such as job strain, physical activity, vitamin C, D, and K levels, and subsequent multivariate analysis in NHANES was also a limitation. Besides, we did not quantify smoking to explore the influence of the amount of smoking on the incidence of OA. However, we demonstrated that smoking increases the risk of OA in current smokers and past smokers based on data from a large population.

## Conclusion

This large national cross-sectional study showed that smoking is positively associated with the prevalence of OA in the US population. It is necessary to further study the relationship between smoking and OA to determine the specific mechanism of smoking on OA.

## Data Availability

The datasets generated during the current study are available in the NHANES repository (http://www.cdc.gov/nchs/nhanes.htm).
